# The Parastyle: A Discrete Trait of the Maxillary Molars

**DOI:** 10.7759/cureus.69733

**Published:** 2024-09-19

**Authors:** Romain Ceinos, Caroline Bernardi, Marie-France Bertrand, Gérald Quatrehomme

**Affiliations:** 1 Odontology, Université Côte d'Azur, Nice, FRA; 2 Institute of Oral and Dental Medicine, CHU de Nice, Nice, FRA; 3 UR 4462 URB2i, Université Paris Cité, Montrouge, FRA; 4 Institute of Forensic Anthropology, Faculty of Medicine, Université Côte d’Azur, Nice, FRA; 5 UMR - CNRS 7264 CEPAM, Université Côte d'Azur, Nice, FRA; 6 UPR 7354 MICORALIS, Université Côte d'Azur, Nice, FRA; 7 Forensic Anthropology, Faculty of Medicine, Université Côte d’Azur, Nice, FRA

**Keywords:** asuda, carabelli cusp, forensic identification, para-molar tubercle, parastyle

## Abstract

This study examines the parastyle, a rare non-metric dental trait observed on the buccal surface of the mesio-buccal cusp of upper molars. Typically unilateral, the parastyle is most frequently found on the second and third maxillary molars, though it occurs at a low frequency. We present a case involving a skull discovered near a stream, where a parastyle was identified on the upper right second molar. This trait, along with other dental findings, was instrumental in building the biological profile of the individual. The rarity of the parastyle enhances its forensic value, offering a useful marker in the identification process, particularly when other identification markers are insufficient or unavailable. This case underscores the potential of rare dental traits in forensic investigations and highlights the importance of incorporating such traits into the broader context of forensic analysis.

## Introduction

A discrete trait, or non-metric trait, refers to an anatomical variation that is typically heritable and exhibits a binary or categorical presence/absence expression. These traits can be found in both bones and teeth, with their frequency varying depending on the population. Some discrete traits are common, while others are rare, making them particularly valuable in anthropology and forensic contexts for population comparisons and individual identification. While discrete traits alone may not always differentiate populations, their distribution and occurrence patterns contribute to constructing the biological profile of individuals, proving useful in both bioarchaeological and forensic investigations [1]. Additionally, some skeletal malocclusions may be associated with specific dental abnormalities or variations, further underscoring the clinical and anthropological significance of these traits [2]. In forensic anthropology, the identification of human remains often relies on the comparison of antemortem (AM) and postmortem (PM) data. Discrete traits, though valuable, should not be used as stand-alone methods for identification. Instead, they serve as complementary evidence to reinforce potential matches or mismatches. When AM records contain information about these anatomical traits, they can help narrow down possible matches and provide additional support to the identification process [3]. However, the utility of these traits depends on their availability and their presence in AM records, which remain the cornerstone of forensic identification. In this paper, we present a case where a parastyle, a rare non-metric trait found on upper molars, was observed on the second maxillary molar of a skull discovered near a stream. The potential role of this rare dental trait in forensic identification is discussed.

## Case presentation

**Figure 1:**
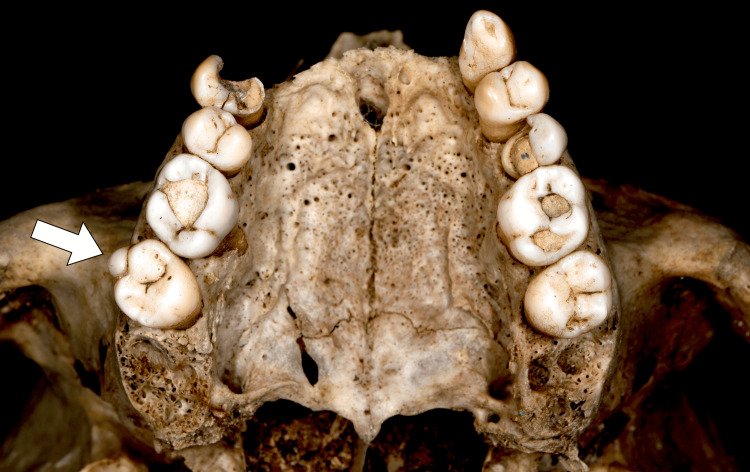
Occlusal view of the maxillary arch from the studied skull. The image highlights the absence of teeth 11, 12, 13, 21, and 22, likely due to a fracture in the anterior maxillary region. Additionally, alveolar resorption is noted on the remaining teeth, indicative of chronic periodontitis. The empty alveoli suggest that teeth 18 and 28 were lost post-mortem. An arrow indicates the parastyle on the buccal surface of tooth 17.

A skull and two femurs were found near a stream and brought to the University Institute of Forensic Anthropology, Faculty of Medicine of Nice (France). Based on these remains, the biological profile suggested a male individual, aged over 50 years, likely between 60 and 70 years old, with a stature of approximately 1.62 m. The remains were estimated to have been several years old. Dental analysis revealed the absence of teeth 11, 12, 13, 21, and 22, likely due to a fracture of the maxillary bone in the anterior region, rather than chronic periodontitis. However, significant alveolar resorption, consistent with periodontitis, was observed on the remaining teeth. The third molars were absent, but the alveolar sockets adjacent to the presumed roots of teeth 18 and 28 suggested a postmortem loss of these teeth (Figure 1).

**Figure 2:**
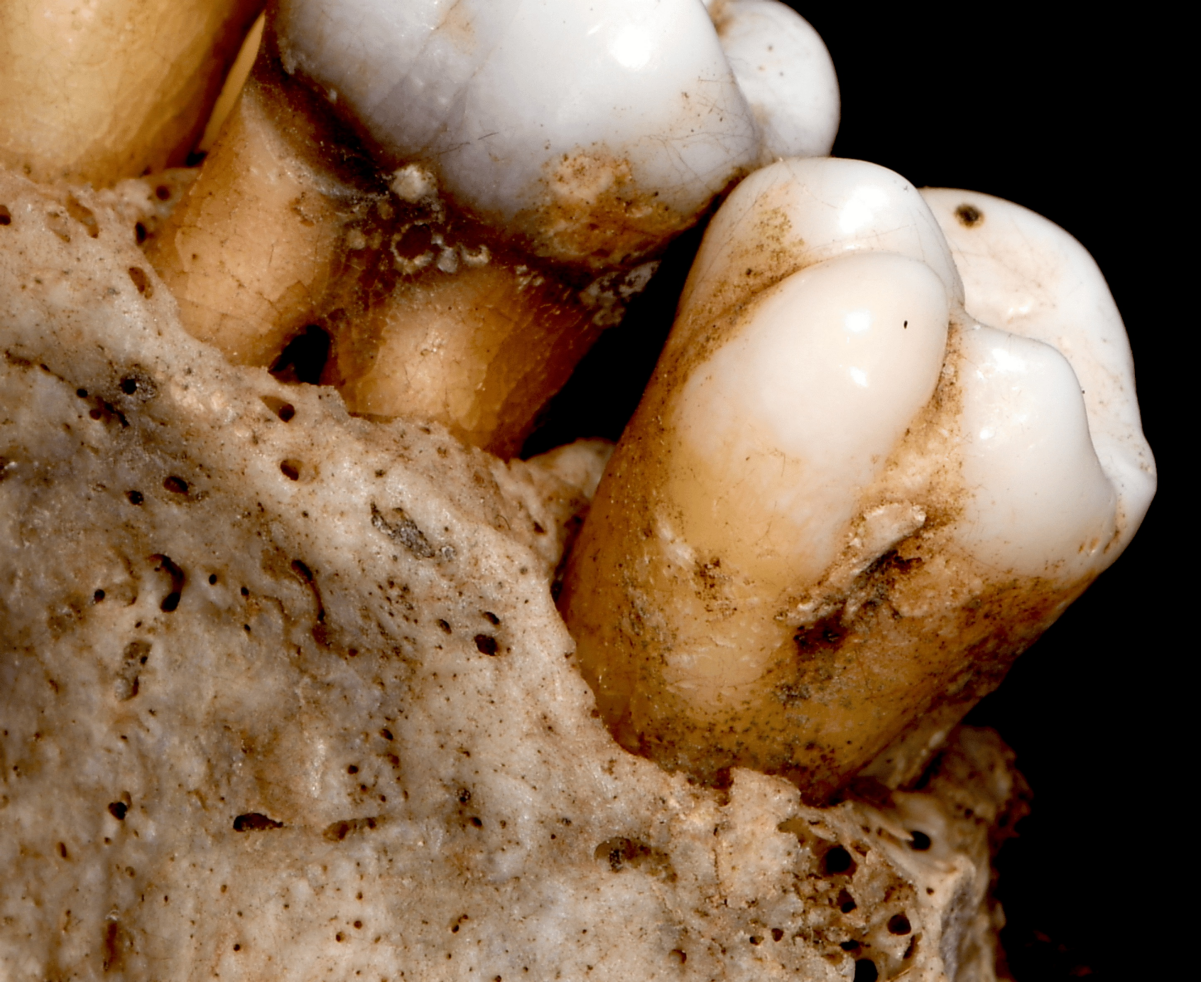
Occluso-buccal view of the right upper second molar (tooth 17) showing a grade 5 parastyle. The large cusp formation extends over the buccal surface of the mesio-buccal cusp, highlighting this rare non-metric dental trait which may have forensic relevance.

**Figure 3:**
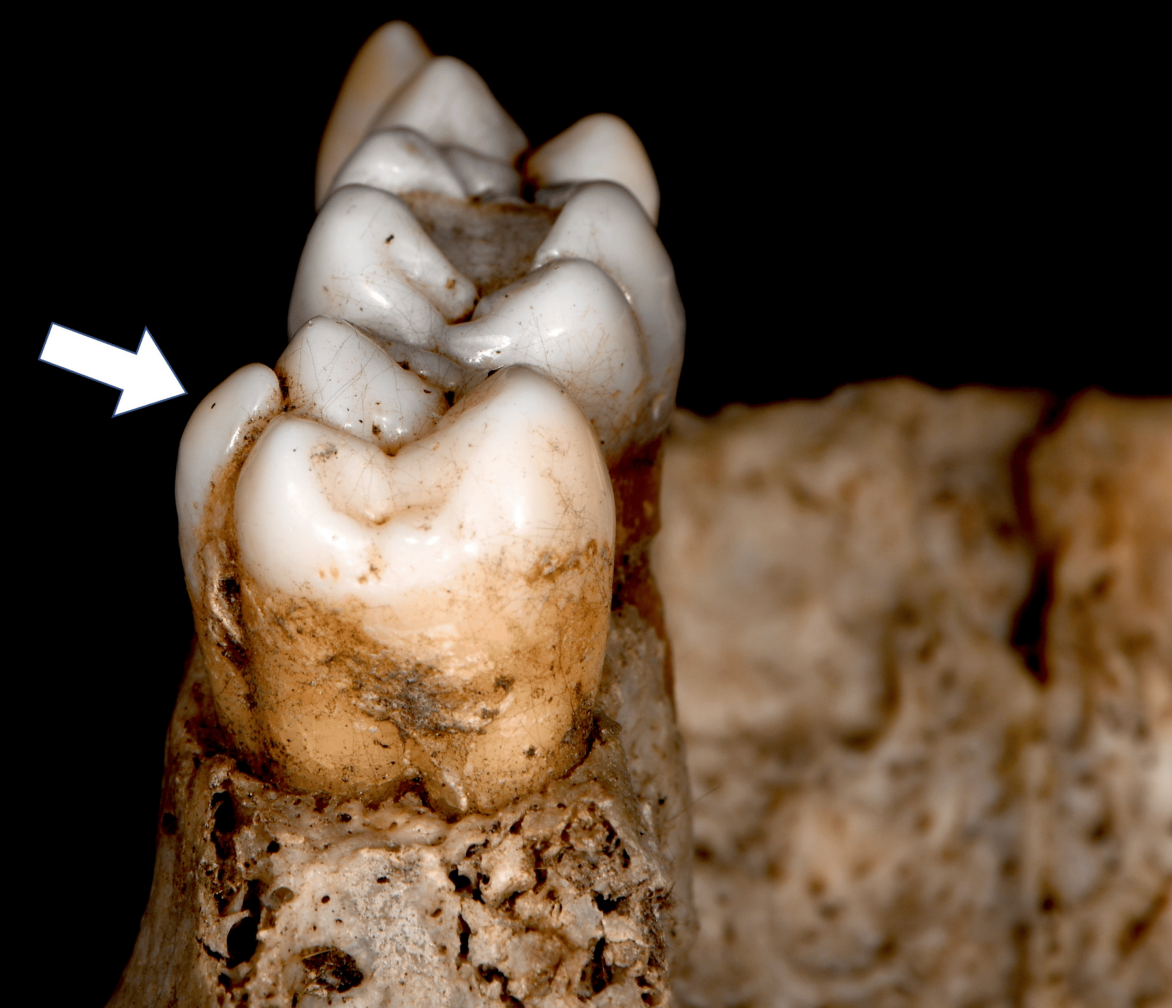
Proximal (distal) view of the right upper second molar (tooth 17) displaying the grade 5 parastyle. The image illustrates the full extension of the parastyle cusp (arrow), with the free apex encroaching on the adjacent disto-buccal cusp. This detailed view helps demonstrate the significant development of the parastyle.

Direct restorative treatments were observed on teeth 16, 25, and 26, along with a large carious lesion on the right maxillary first premolar. Additionally, a prominent tubercle was found on the buccal surface of tooth 17, identified as a non-metric dental trait known as a parastyle (Figures 2, 3). While the individual remains unidentified, the detailed description of this rare dental trait adds to the growing body of knowledge on non-metric traits. This case aims to assist the scientific community in future identifications by documenting and emphasizing the forensic relevance of such rare traits.

## Discussion

There are many discrete dental traits, encompassing both crown and root features. However, the terminology surrounding these traits can sometimes be confusing due to the various synonyms used in the literature. Systems such as the Arizona State University Dental Anthropology (ASUDA) system [4] have standardized the classification and interpretation of these characteristics. These morphological traits, which vary in expression, are commonly used by anthropologists to compare ancient and contemporary human populations. They are typically hereditary, stable, and exhibit little to no sexual dimorphism.

The parastyle is a tubercle located on the buccal surface of the mesio-buccal cusp of the upper molars. In its most pronounced form, it can extend onto the disto-buccal cusp. This discrete trait is usually unilateral and is most frequently found on the second and third maxillary molars, with its occurrence on the first molar being extremely rare (less than 0.1%). The presence of the parastyle on the second and third maxillary molars ranges between 2.8% and 4.7% [5], with frequencies in the literature consistently below 10% [6]. In some cases, the parastyle may be connected to an endodontic canal shared with other root canals [7-9]. This structure originates from the vestibular cingulum, suggesting it may be a vestige of molars found in lower primates [10]. Due to its location, this anatomical feature is prone to periodontal and carious pathology, as it tends to retain plaque [11, 12].

Several classification systems for the parastyle have been proposed in the literature [4], ranging from grade 0 (absence) to grade 5 (a large, free-standing cusp that may extend over the paracone and metacone). It is important to distinguish the parastyle from other additional tubercles or cusps, such as the paramolar tubercle, which can be present on both maxillary and mandibular molars [13, 14]. The parastyle, however, is unique to the upper molars. Often regarded as the "mirror image" of the Carabelli cusp, found on the palatal surface of the mesio-palatal cusp of the maxillary molars, the parastyle holds particular significance for identification purposes [15].

## Conclusions

The parastyle is a rare, non-metric dental trait with limited application in population studies due to its consistently low frequency. However, its rarity enhances its importance in forensic identification, particularly in cases where other identification markers are unavailable or insufficient. The observation of such a distinctive feature, along with other dental and skeletal indicators, can contribute to building a detailed biological profile, as illustrated in this case. Though uncommon, the presence of the parastyle on maxillary molars underscores the value of comprehensive dental analysis in both forensic anthropology and dental anthropology. Future research should further investigate the forensic relevance of rare dental traits like the parastyle across various populations.
